# Recurrent angiofibroma: analysis of risk factors and common sites of recurrence

**DOI:** 10.1007/s00405-025-09476-9

**Published:** 2025-06-03

**Authors:** Hisham Mohamed Anwar Attya, Mohamed Salah Hassouna, Abdelrahman Ali Shawky, Mena Esmat Abdelmalek

**Affiliations:** Cairo University Kasr Alainy Faculty of Medicine, Cairo, Egypt

**Keywords:** Juvenile nasopharyngeal angiofibroma, Recurrence, Risk factors

## Abstract

**Objective:**

To detect risk factors and common anatomical sites for recurrence of juvenile nasopharyngeal angiofibroma (JNA).

**Methods:**

This is a retrospective study, included all male patients who were diagnosed histopathologically with juvenile nasopharyngeal angiofibroma (JNA) and were operated before at Kasr Al-Ainy Hospital, Cairo University in the period between January 2012 and December 2021. Their clinical data were retrieved and analyzed.

**Results:**

Among 68 patients included in this study, 26 patients experienced recurrence with total recurrence rate (38.2%). JNA recurrence was significantly associated with primary tumor size (≥ 4 cm), advanced primary tumor stages (stage IIIa, IIIb according to Radkowski classification) and their correlatives of preoperative embolization, perioperative blood transfusion or open surgical approach. Age on presentation, tumor stage, perioperative blood transfusion and tumor size were significant factors affecting the recurrence rate according to cox regression univariate analysis, while on multivariate analysis the only significant independent predictors of JNA recurrence were age on presentation and tumor size. 61.53% of recurrent cases were discovered accidently through their regular postoperative follow up examination and/or imaging and were asymptomatic upon diagnosis of recurrence. Moreover, patients with early tumor stage or didn’t undergo preoperative embolization or had a primary tumor size < 4 cm had significantly longer recurrence interval than those with advanced tumor stage or underwent preoperative embolization or had a primary tumor size ≥ 4 cm. Pterygoid process (92.3%) was significantly the commonest to be invaded by recurrent/residual tumor, followed by nasopharynx (84.6%) and sphenoid sinus (76.9%).

**Conclusion:**

JNA recurrence is significantly associated with primary tumor size, primary tumor stage and their correlatives of preoperative embolization, perioperative blood transfusion or open surgical approach, but age on presentation and primary tumor size were the only independent predictors of tumor recurrence. Meticulous surgical attention should be paid for pterygoid process in order to decrease residual/recurrence incidence.

## Introduction

Juvenile nasopharyngeal angiofibroma (JNA) is a rare vascular tumor that arises only in adolescent males [1]. Despite its benign nature, JNA has a high predilection for bone destruction and local invasion. Sphenopalatine foramen is thought to be the primary origin of the tumor and from there it can spread towards multiple anatomical locations at the same time including the nasal cavity, nasopharynx, paranasal sinuses, orbit, pterygopalatine fossa, infratemporal fossa and intracranial cavity [2].

Histologically, JNA is composed of fibrous connective tissue stroma rich in vasculature which is lined by a single layer of endothelial cells and occasional smooth muscle without a true tumor capsule. Deficient muscular layer in the tumor vasculature and absence of stromal elastic fibers gives rise to recurrent episodes of severe bleeding and complicates surgical tumor excision [3].

Classic clinical tumor presentation includes recurrent unilateral epistaxis and nasal obstruction; however, more advanced disease stages can present with facial swelling, proptosis and diplopia. Diagnosis is based on endoscopic examination and imaging studies (computed tomography [CT] and magnetic resonance imaging [MRI]) while biopsy is not recommended to avoid severe bleeding [4].

Although multiple modalities have been adopted for JNA treatment including steroids, estrogen, anti-androgens or radiotherapy; surgical excision remains the standard treatment. Many surgical approaches have been used, but recently the endoscopic approach has become the standard surgical technique due to less morbidity and better visualization of the surgical field [5]. Along past decades, JNA recurrence represented one of the greatest obstacles for otolaryngologists despite effective surgical treatment. Multiple studies have suggested that JNA recurrence could be affected by the clinical and pathological characteristics of the disease. Hence, clinical risk factors should be explored for JNA recurrence prediction and rate reduction [6].

## Materials and methods

This retrospective study was concerned with all patients diagnosed clinically and histopathologically with juvenile nasopharyngeal angiofibroma (JNA) who were operated before at Kasr Al-Ainy Hospital, Cairo University in the period between January 2012 and December 2021. Patients not proved to be angiofibroma histopathologically after primary tumor excision or those with incomplete medical records were excluded.

The Research Ethics Committee of the Faculty of Medicine, Cairo University approved this study on 29-8-2022 under the code of MD-221-2022.

**Fig. 1:**
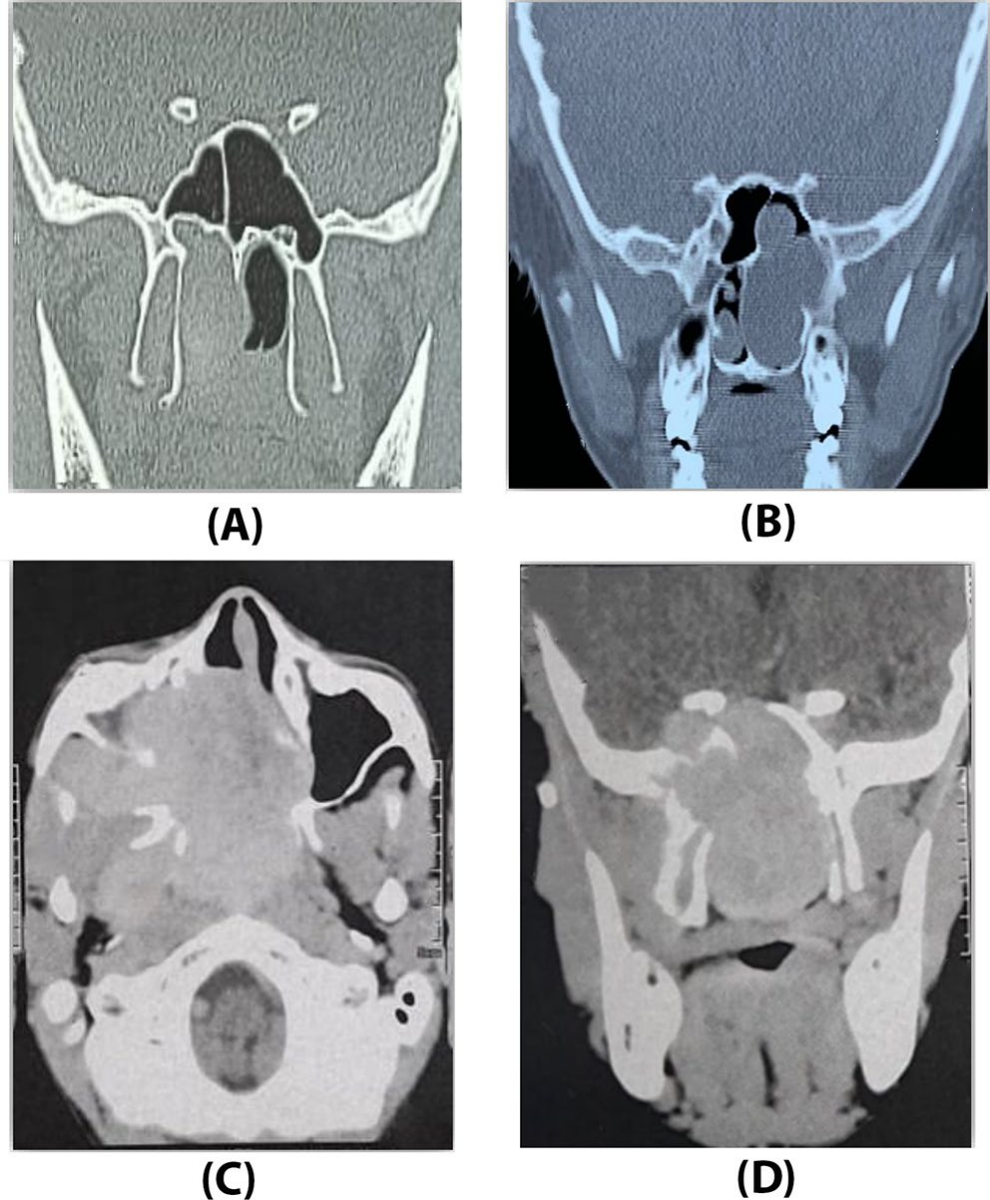
Examples of pre-operative imaging of juvenile nasopharyngeal angiofibroma (JNA): (**A**) stage Ia limited to nasopharynx and nasal cavity. (**B**) stage Ib extending to sphenoid sinus. (**C**) stage IIc extending to pterygopalatine and infratemporal fossae (**D**) stage IIIa with intracranial extension through the sphenoid sinus

Patients’ radiological data (CT ± MRI) and operative details were retrieved and analyzed. Recurrent juvenile nasopharyngeal angiofibroma (JNA) was defined as tumor presence in the nasopharynx or neighboring structures demonstrated clinically and endoscopically and confirmed by follow up imaging after the first surgical treatment. The included patients’ data were retrieved from medical records in the form of:

(I) **History:** Including age at the first presentation, chief complaint of the patient on diagnosis with primary and recurrent tumor, duration between first operation and diagnosis of tumor recurrence and other associated symptoms encompassing: nasal obstruction, recurrent epistaxis, discharge, headache, ocular and aural symptoms.
(II) **ENT examination:** Focusing on tumor extensions of both primary and recurrent tumors revealed by nasal endoscopy.
(III) **Radiological data:**
  A. Primary tumor size and tumor extensions in CT with contrast or MRI were reported.
  B. Staging of the primary tumor was done according to “Radkowski classification” being simple and widely used (Fig. 1).
(IV) **Surgical details:** including preoperative angiography (feeding vessels, embolization), history of blood transfusion and surgical approach.

### Statistical analysis

All collected data was revised for completeness and accuracy. Pre coded data was entered on the computer using the statistical package of social science software program, version 26 (SPSS) to be statistically analyzed. Statistical analysis was performed with IBM SPSS ver. 22.0 (IBM Corp., Armonk, NY, USA) and GraphPad Prism 7 (San Diego, CA, USA) and Microsoft Excel 2016.

All quantitative data (like time free interval before tumor recurrence) were expressed as mean ± standard deviation (SD). All qualitative data (such as different tumor stages or presenting symptoms or sites of recurrence) were presented as frequency and percentages and presented in tables & graphs. Chi-square and Fisher’s exact tests were performed to compare the influence of clinicopathological features between patients with and without recurrence. Then, risk factors for JNA recurrence were analyzed by univariate and multivariate analyses using Cox hazard regression model. P values less than or equal to 0.05 were considered statistically significant.

## Results

This study included 68 patients diagnosed with JNA who were operated primarily at Kasr Al-Ainy Hospital, Cairo University in the period between January 2012 and December 2021. They were exclusively males (100%) with mean age about (15.3 ± 4.2 years) (range 5–24 years). 26 patients experienced recurrence with total recurrence rate (38.2%). General patient and tumor characteristics are summarized in (Table 1).

### Interval to recurrence

The mean interval to recurrence among patients was (18.28 ± 14.97) months, (57.7%) of patients were diagnosed with recurrent tumor in the first year after primary tumor excision, while (15.4%) were diagnosed in the second year, (15.4%) in the third year, (7.7%) in the fourth year and (3.8%) in the fifth year.

### Symptoms of recurrence

We reported that (15.38%) of patients presented with proptosis, (11.53%) presented with recurrent epistaxis, (11.53%) presented with nasal obstruction, while (61.53%) were asymptomatic when they were diagnosed as recurrent cases denoting that majority of cases were discovered accidently through their regular postoperative follow up examination and/or imaging.

### Risk factors for recurrence

Recurrence rate among patients aged < 15 years (50%) was higher than patients aged ≥ 15 (28.9%). However, this difference wasn’t statistically significant (*P* = 0.07).

Recurrence rate was higher among patients presented primarily with facial swelling (83.3%) and/or proptosis (87.5%) with statistically significant association.

Recurrence rate among patients with stage III primary tumor (71.5% in stage IIIa and 100% in stage IIIb) was higher than patients with stage I primary tumor (20% in stage Ia and 25% in stage Ib). This difference was highly statistically significant (*P* = 0.01).

Recurrence rate among patients who underwent preoperative embolization (41.4%) was higher than patients who were operated without preoperative embolization (20%). This difference was highly statistically significant (*P* = 0.0001).

Recurrence rate among patients who needed perioperative blood transfusion (56.5%) was higher than patients who didn’t need perioperative blood transfusion (28.9%). This difference was highly statistically significant (*P* = 0.02).

Recurrence rate among patients who were operated through open surgical approach (83.3%) was higher than patients who were operated through endoscopic surgical approach (33.9%). This difference was highly statistically significant (*P* = 0.01).

Recurrence rate among patients who had a primary tumor sized ≥ 4 cm (54.1%) was higher than patients who had a primary tumor sized < 4 cm (19.4%). This difference was highly statistically significant (*P* = 0.003).

**Table 1:** Summary of patients’ characteristics and risk factors for recurrence

			All		Recurrence		P value
			N	%	N	%	
Age range (15.3 ± 4.2 years)		< 15	30	44.1%	15	50.0%	0.07
		≥ 15	38	55.9%	11	28.9%	
Symptoms of primary tumor	Nasal obstruction	No	0	0.0%	0	0.0%	-----
		Yes	68	100.0%	26	38.2%	
	Recurrent epistaxis	No	29	42.6%	8	27.6%	0.11
		Yes	39	57.4%	18	46.2%	
	Facial swelling	No	62	91.2%	21	33.9%	0.01*
		Yes	6	8.8%	5	83.3%	
	Proptosis	No	60	88.2%	19	31.7%	0.002*
		Yes	8	11.8%	7	87.5%	
	Hyposmia	No	66	97.1%	24	36.4%	0.06
		Yes	2	2.9%	2	100.0%	
Stage		Ia	10	14.7%	2	20.0%	0.01*
		Ib	8	11.8%	2	25.0%	
		IIa	12	17.6%	2	16.7%	
		IIb	10	14.7%	4	40.0%	
		IIc	16	23.5%	6	37.5%	
		IIIa	7	10.3%	5	71.4%	
		IIIb	5	7.4%	5	100.0%	
Embolization		No	10	14.7%	2	20%	0.0001*
		Yes	58	85.3%	24	41.4%	
Blood transfusion		No	45	66.2%	13	28.9%	0.02*
		Yes	23	33.8%	13	56.5%	
Surgical approach		Endoscopic	62	91.2%	21	33.9%	0.01*
		Open	6	8.8%	5	83.3%	
Tumor size		< 4	31	45.6%	6	19.4%	0.003*
		≥ 4	37	54.4%	20	54.1%	

### Predictors of postoperative tumor recurrence: Cox regression analysis

The univariate analysis of risk factors of JNA recurrence revealed that age on presentation, tumor stage, perioperative blood transfusion and tumor size were significant factors affecting the recurrence rate while on multivariate analysis the only significant independent predictors of JNA recurrence were age on presentation and tumor size where the younger age showed higher hazard to develop recurrence than older (HR & 95th C.I. is 0.914(0.837–0.999)) and the larger tumour size (< 4 cm) is 4 times associated with increase in the recurrence rate (HR & 95th C.I. is 4.442(1.432–13.778)) (Table 2).

**Table 2:** Univariate and multivariate Cox hazard regression analysis of the risk factors of JNA recurrence

	Univariate		Multivariate	
Variables in the Equation	**P value**	**HR (95th C.I.)**	**P value**	**HR (95th C.I.)**
Age on presentation	0.013*	0.889(0.811–0.975)	0.047*	0.914(0.837–0.999)
Tumor stage	< 0.001*	4.691(2.103–10.466)	0.104	3.213(0.788–13.105)
Embolization	0.231	2.421(0.57–10.27)	…	…
Blood transfusion	0.031*	2.333(1.079–5.041)	0.239	0.443(0.114–1.717)
Tumor size	0.006*	3.608(1.444–9.014)	0.01*	4.442(1.432–13.778)

### Sites of recurrence

**Fig. 2:**
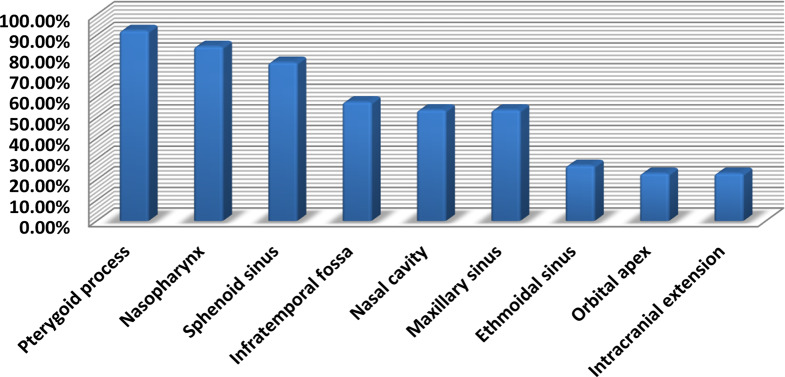
Column chart showing sites of recurrence

Frequency and percentages of different sites regarding recurrent tumor were presented in (Table 3) and (Fig. 2). Statistical comparison between different sites of recurrence was performed by using Chi square test which revealed that pterygoid process (92.3%) was significantly the commonest to be invaded by recurrent/residual tumor, followed by nasopharynx (84.6%) and sphenoid sinus (76.9%).

**Table 3:** Common sites of recurrent tumor and their percentage

	*N*	%
Pterygoid process	24	92.3%
Nasopharynx	22	84.6%
Sphenoid sinus	20	76.9%
Infratemporal fossa	15	57.7%
Nasal cavity	14	53.8%
Maxillary sinus	14	53.8%
Ethmoidal sinus	7	26.9%
Orbital apex	6	23.1%
Intracranial extension	6	23.1%

### Relation between recurrence interval and risk factors

Mean and standard deviation of time free interval before tumor recurrence (month) among different categories of age range, primary tumor stage, preoperative embolization, perioperative blood transfusion, surgical approach, and primary tumor size were presented in (Table 4).Statistical comparison was done between different categories (Independent t test to compare between 2 categories / One Way ANOVA test to compare between more than 2 categories) which revealed that there were a significant association between recurrence interval and primary tumor stage (*P* < 0.0001), preoperative embolization (*P* < 0.0001) and primary tumor size (*P* < 0.0001).

**Table 4:** Recurrence interval (Mean ± SD) among patients

		Recurrence interval			
		N	Mean (16.7 ± 14.9 months)	± Standard Deviation	P value
Age	< 15	30	16	14	0.61
	> or = 15	38	18	17	
Stage	Ia	10	42	8	< 0.0001*
	Ib	8	42	25	
	IIa	12	9	4	
	IIb	10	8	4	
	IIc	16	18	12	
	IIIa	7	8	9	
	IIIb	5	14	10	
Embolization	No	10	31	21	< 0.0001*
	Yes	58	12	10	
Blood transfusion	No	45	22	18	0.01*
	Yes	23	12	9	
Surgical approach	Endoscopic	62	18	16	0.37
	Open	6	12	11	
Tumor size	< 4	31	31	21	< 0.0001*
	≥ 4	37	12	10	

## Discussion

Juvenile nasopharyngeal angiofibroma (JNA) is a highly vascular tumor of the nasopharynx that develops exclusively in adolescent males. It is a relatively rare neoplasm, accounting for 0.05–1% of all head and neck tumors, but its treatment is still challenging due to local aggressive behaviour of the tumor and the associated risk of intra-operative profuse bleeding during surgical excision favoring for high rate of recurrence [7, 8]. Due to rarity of the disease, making prospective, randomized double-blind analysis studies is very difficult. Therefore, case report/series, meta-analysis and systematic review studies were dominant in the literature and provide valuable information when these optimal studies are not feasible [1].

In this study, we retrospectively reviewed 68 patients diagnosed with juvenile nasopharyngeal angiofibroma, 26 of them experienced recurrence with total recurrence rate (38.2%). The mean interval to recurrence among patients was (16.7 ± 14.9) months, 15 patients were diagnosed with recurrent tumor in the first year after primary tumor excision (57.7%). Sun et *al.* retrospectively reviewed records of 97 cases and (39.2%) of them experienced JNA recurrence of which 17 recurrences (44.7%) occurred within 12 months of the initial surgery [9]. Janakiram et al. retrospectively reviewed records of 242 primary JNA cases. All patients were evaluated with postoperative contrast enhanced CT within 36 h. of surgery to identify any tumor residuals. Hence, they differentiated between residual and recurrent tumors and redefined tumor recurrence more accurately as new tumor detected in the follow up imaging studies after an initially negative postoperative CT scan. So, they reported 19 cases with residual disease (7.8%) and 22 with recurrent disease (9%) as different categories [10].

Majority of cases presented primarily with nasal obstruction and recurrent epistaxis (100% and 57.4% respectively) with no statistically significant association with tumor recurrence. On the other hand, (83.3%) and (87.5%) of patients who presented with facial swelling or proptosis respectively experienced JNA recurrence which represented a statistically significant association. This can be interpreted with the coincidence of facial swelling and proptosis with infratemporal and intraorbital tumor extension which represent more advanced tumor stage. Among patients with recurrent tumor, 61.53% were discovered accidently through their regular postoperative follow up examination and/or imaging and were asymptomatic upon diagnosis of recurrence. So, early postoperative imaging is recommended to exclude any residual deposits and to differentiate it from true recurrence. Regular postoperative follow up with nasal endoscopy is also recommended to detect any asymptomatic recurrence which will need closer follow up and may be indicated for revision surgery to abort any further tumor progression.

Pamuk et al. reviewed records of 48 JNA cases retrospectively. The most common presenting symptom in their cases was nasal obstruction (91.6%), followed by recurrent epistaxis (83.3%). So, they attributed the high percentage of patients presented with advanced stage tumors to the misdiagnosis of chronic nasal obstruction as having adenoid hypertrophy or rhinosinusitis, and not performing nasal endoscopy especially in those with recurrent epistaxis [11].

Although patients aged < 15 years in this study had a higher recurrence rate (50%) than those who aged ≥ 15 years (28.9%), There was no statistically significant association between age range and recurrence (*P* = 0.07). Liu et al. reported also no statistically significant association between age range and recurrence as the recurrence rate was 16.2% among patients aged ≤ 18 years and 18.6% among patients aged > 18 years [12]. In contrary, Pamuk et al. reported that patients who were diagnosed at age less than 14 years had a significant higher recurrence rate (34.7%) in comparison with those who were diagnosed at age more than 14 years (8%) [11].

In this series, the highest recurrence rate was reported among stage III patients according to Radkowski classification (71.5% in stage IIIa and 100% in stage IIIb) with a highly significant association between primary tumor stage and recurrence (*P* = 0.01). Pamuk *et* al. reported that highest recurrence rate among patients with Radkowski stage IIIB tumors (100%) and Önerci stage IV tumors (66.6%). In contrast, no recurrence was reported among patients with Radkowski stage I–IIB or Önerci stage I–II tumors [11].

We reported that there was a highly significant association between preoperative embolization and recurrence (*P* = 0.0001) as 41.4% of patients who underwent preoperative embolization experienced recurrence versus only 20% of patients who were operated without preoperative embolization. Endoscopic excision after preoperative embolization especially in advanced stage disease was the standard management at our institute in the period included in the study and that can interpret this result and rebut the impression that preoperative embolization is a direct risk factor that increase tumor recurrence. Moreover, preoperative embolization was revealed to be insignificant predictor of tumor recurrence in the Cox regression analysis which coincides with the former interpretation.

McCombe et al. reported in their case series that preoperative embolization is associated with both rapid and frequent recurrence. So, they assumed that embolization shrinks the tumor but in the same time makes complete removal more difficult with larger amount of residual tissue [13]. In contrary, Diaz et al. in their systematic review compared outcomes of juvenile nasopharyngeal angiofibroma (JNA) resection between embolized and non embolized cohorts, and between transarterial embolization (TAE) and direct puncture embolization (DPE). They found that patients undergoing embolization were less likely to experience recurrence. So, they hypothesized that pre-operative embolization can decrease tumor recurrence as it facilitates excision of a greater portion of the tumor due to improved intraoperative visualization and reduced incidence of uncontrolled bleeding [14].

We also found that there was a highly significant association between perioperative blood transfusion and recurrence (*P* = 0.02) as 56.5% of patients who needed perioperative blood transfusion experienced recurrence versus only 28.9% of patients who didn’t need perioperative blood transfusion. Blood transfusion reflects more severe intraoperative blood loss that can worsens field visualization, affects radicality of tumor excision and leads directly to more tumor residuals. There was a consensus between Liu et al. and Fang et al. in a result which revealed that patients who had an intraoperative blood loss ≥ 800 mL were associated significantly with higher recurrence rate (31.6% and 68.18% respectively) more than those who had an intraoperative blood loss < 800 mL (5.4% and 31.82% respectively) [6, 12].

There was a highly significant association between surgical approach and recurrence (*P* = 0.01) as 83.3% of patients who were operated through open surgical approach experienced recurrence versus only 33.9% of patients who were operated through endoscopic surgical approach. In the period included in the study, endoscopic excision of the tumor became the standard surgical approach at our institute even with advanced stage tumors and open approaches were preserved only for more advanced tumors which were thought to be hardly managed with endoscopic approach. Hence was the major difference in recurrence rate between both approaches. Boghani et al. compared in their systematic review (in the period between 1990 and 2012) between endoscopic, endoscopic assisted and open approaches in 1047 patients with JNA and concluded that there is no strong evidence that one approach have a privilege over the other regarding recurrence rate (range 5–50%) [1]. But Reyes et al. found in their meta-analysis that the endoscopic approach has a lower chance of tumor recurrence when compared with open approaches (18% and 28% respectively) [4].

In this series, there was a highly significant association between primary tumor size and recurrence (*P* = 0.01) as 54.1% of patients who had a primary tumor sized ≥ 4 cm experienced recurrence versus only 19.4% of patients who had a primary tumor sized < 4 cm. This could be attributed to complexity of JNA spread to multiple anatomical sites with increasing tumor size which affect complete resectability of the tumor and favors for more tumor residuals. Fang et al. reported that patients with a primary tumor sized > 4 cm experienced significantly higher recurrence rate (81.82%) compared with patients who had a primary tumor sized ≤ 4 cm (18.18%) [6].

The univariate Cox hazard regression analysis revealed that JNA recurrence was affected by age on presentation, tumor stage, perioperative blood transfusion and tumor size while on multivariate analysis the only significant independent predictors of JNA recurrence were age on presentation and tumor size where the younger age showed higher hazard to develop recurrence than older (HR & 95th C.I. is 0.914(0.837–0.999)) and the larger tumour size (< 4 cm) is 4 times associated with increase in the recurrence rate (HR & 95th C.I. is 4.442(1.432–13.778)).

Fang et al. reported in their univariate Cox regression model that year at diagnosis (before 2010), tumor size (< 4 cm), sphenoid bone invasion, Radkowski stage, surgical approach (open or combined endoscopic and open surgery) and intraoperative bleeding (≥ 800 mL) were factors affecting JNA recurrence. Further multivariable analysis showed that year at diagnosis, sphenoid bone invasion and surgical approach were independent predictors of JNA recurrence [6].

Statistical comparison between groups of different risk factors and their mean recurrence interval was done and revealed that there was a significant association between recurrence interval and primary tumor stage (*P* < 0.0001), preoperative embolization (*P* < 0.0001) and primary tumor size (*P* < 0.0001). Patients with early tumor stage or didn’t undergo preoperative embolization or had a primary tumor size < 4 cm had significantly longer recurrence interval than those with advanced tumor stage or underwent preoperative embolization or had a primary tumor size ≥ 4 cm.

Fang et al. reported that patients who were diagnosed after 2010, with early Radkowski stage disease, received endoscopic surgery and had intraoperative bleeding < 800 mL had longer recurrence-free survival [6].

Comparison between different sites of recurrence was performed and revealed that pterygoid process (92.3%) was significantly the commonest to be invaded by recurrent/residual tumor, followed by nasopharynx (84.6%) and sphenoid sinus (76.9%).This result can reflect the invasive character of JNA that can spread through bony diploe, canals and fissures. So, meticulous drilling of pterygoid process and basisphenoid should be routinely done to decrease residual/recurrence incidence. Janakiram et al. reported 19 cases with residual disease and 22 recurrent cases during the early phase of surgical experience with endoscopic resections and encountered that residual and recurrent tumors most commonly located in the pterygoid wedge as a total of 10 recurrences occurred in this area. In the subsequent cases (which were performed with more endoscopic experience), the cancellous bone of the pterygoid wedge was drilled away to ensure complete tumor removal, which allowed significant reduction in the incidence of residual and recurrent disease. With more evolvement in surgical expertise, more advanced tumors were endoscopically resected and tumor recurrences were noticed only in the superior orbital fissure, quadrangular space, inferior orbital fissure, and surrounding internal carotid artery (ICA) [15].

This study had some limitations like the discrepancy in number of patients regarding surgical approach (endoscopic or open) and preoperative embolization (whether done or not) which affected the accuracy of clear association between these factors and tumor recurrence. Restricted study population was another limitation due to rarity of the disease so that all patients operated along wide period of time were included. So, we recommend future multicentric studies which will offer larger study population. Being retrospective study was another limitation as it may lead to selection bias. Finally, early postoperative imaging wasn’t available in most of patients’ data making it difficult to differentiate obviously between residual and recurrent disease.

## Conclusion

JNA recurrence is significantly associated with primary tumor size, primary tumor stage and its correlatives of preoperative embolization, perioperative blood transfusion or open surgical approach, but age on presentation and primary tumor size were the only independent predictors of tumor recurrence. Pterygoid process was the commonest site to be invaded by recurrent/residual tumor and meticulous surgical attention should be paid for it in order to decrease residual/recurrence incidence.
